# Efficacy of physical and chemical disinfection against clinically relevant free-living amoebae isolated from drinking water and plumbing biofilms

**DOI:** 10.3389/fmicb.2025.1654984

**Published:** 2025-11-28

**Authors:** Muhammad Atif Nisar, Claire Hayward, Kirstin E. Ross, Melissa H. Brown, Richard Bentham, Harriet Whiley

**Affiliations:** 1 College of Science and Engineering, Flinders University, Bedford Park, SA, Australia; 2 Cooperative Research Centre for Solving Antimicrobial Resistance in Agribusiness, Food and Environments (CRC SAAFE), Future Industries Institute, University of South Australia, Mawson Lakes, SA, Australia; 3 ARC Training Centre for Biofilm Research and Innovation, Flinders University, Bedford Park, SA, Australia

**Keywords:** protozoa, opportunistic pathogens, potable water, infection control, biofilm

## Abstract

**Introduction:**

Free-living amoebae (FLA) are commonly found in drinking water systems and biofilms, posing public health risks both as pathogens and as hosts for opportunistic bacteria such as Legionella spp. and Mycobacterium spp. Current water management practices are often insufficient to control biofilm-associated FLA and their intracellular pathogens.

**Methods:**

This study evaluated the efficacy of multiple disinfectants—thermal disinfection (70 °C), hydrogen peroxide, 2-methyl-4-isothiazolin-3-one, benzalkonium chloride (BAC), sodium hypochlorite, and chlorine dioxide—against planktonic trophozoites and cysts of five FLA strains: Acanthamoeba DB1, Allovahlkampfia DS1, Stenamoeba DS1, Vermamoeba vermiformis HB7, and Acanthamoeba polyphaga ATCC^®^ 30461^™^. Disinfectants were tested at varying concentrations and exposure times.

**Results:**

Thermal disinfection was the most effective treatment, followed by hydrogen peroxide. Exposure to 70 °C for 45 min achieved >4 log_10_ reduction of all FLA trophozoites. Thermal treatment at 70 °C for 60 min produced >4 log_10_ reduction for all cysts except V. vermiformis HB7, which showed greater thermal tolerance.

**Discussion:**

The findings demonstrate that thermal disinfection is a highly effective strategy for inactivating multiple FLA species in their trophozoite and cyst forms. Further research is needed to evaluate the feasibility and effectiveness of applying these strategies to control biofilm-associated FLA within plumbing fixtures and drinking water distribution systems.

## 1 Introduction

FLA, also known as naked amoebae, are aerobic amoeboid microorganisms that move using pseudopodia (Page, 1988; Smirnov and Goodkov, 1999). These eukaryotic microbes can exist in two different physiological states: the trophozoite, a metabolically active vegetative form, and cysts a highly resistant dormant form (Critchley and Bentham, 2009; Smirnov et al., 2011; Dupuy et al., 2014). FLA are ubiquitously distributed in all types of natural and artificial environments, including potable water distribution systems and plumbing biofilms (Page, 1988; Thomas and Ashbolt, 2011; Potgieter et al., 2021; Malinowski et al., 2022; Samba-Louaka, 2023; Ariyadasa et al., 2024; Vingataramin et al., 2025). Some of these FLA, such as *Acanthamoeba*, *Naegleria*, and *V. vermiformis* (formerly known as *Hartmannella vermiformis*) found in these water distribution systems, are directly or indirectly associated with various human diseases and pose public health risks (Visvesvara et al., 2007; Boamah et al., 2017; Nisar et al., 2020b). These diseases include amoebic keratitis, particularly among contact lens users, with cases due to *Acanthamoeba* spp. increasing steadily each year. This trend is concerning given that *Acanthamoeba* are frequently detected in drinking water distribution systems and can serve as a source of exposure. Poor hygiene practices, such as the failure to comply with recommended lens cleaning and disinfection procedures, and the rinsing and storing of lenses in non-sterile saline or tap water, are recognized risk factors for infection (Larkin et al., 1990; Kilvington et al., 2004). Other associated diseases include cutaneous and pulmonary infections and fatal granulomatous amoebic encephalitis in both immunocompromised and immunocompetent individuals (Rayamajhee et al., 2021; Petrillo et al., 2024). While *Naegleria fowleri* causes primary amoebic meningoencephalitis with a > 95% case fatality rate (Heggie and Küpper, 2017), it was not included in this study; instead, the focus is on FLA more commonly associated with biofilms in potable water systems, such as *Acanthamoeba* and *Vermamoeba*, which are also relevant to ocular infections. These health risks, particularly for vulnerable populations, highlight the importance of controlling FLA in potable water distribution systems.

FLA can also be carriers, reservoirs, and vectors of opportunistic premise plumbing pathogens (OPPPs) such as certain members of the *Legionella*, *Mycobacterium,* and *Pseudomonas* genera (Thomas and Ashbolt, 2011; Boamah et al., 2017). In engineered water systems and building plumbing systems, the trophozoites support intracellular growth and propagation of OPPPs, whereas cysts protect intracellular OPPPs from prolonged chemical and physical water disinfection procedures (Thomas et al., 2010; Nisar et al., 2020a). The presence of FLA has also suggested to increase the number and virulence of certain pathogenic bacteria capable of intracellular replication, such as pathogenic *Legionella* spp. and *Mycobacterium* spp. (Ashbolt, 2015). Within engineered potable water systems, *V. vermiformis* and *Acanthamoeba* are the most abundant and important FLA associated with OPPPs such as *Legionella* (Thomas et al., 2010; Nisar et al., 2020a; Nisar et al., 2022; Hayward et al., 2025). A recent study conducted in Australia found that the presence of *L. pneumophila* in potable water or biofilm samples was always associated with the presence of an amoebic host (Nisar et al., 2022). As such, future disinfection strategies targeting OPPPs should account for amoebic hosts, such as *V. vermiformis* and *Acanthamoeba,* that are highly resistant to disinfection treatments and environmental stressors (Storey et al., 2004; Dupuy et al., 2011; Dupuy et al., 2014; Rhoads et al., 2015; Dobrowsky et al., 2016; He et al., 2021).

Worldwide, potable water disinfection guidelines have been specifically designed to control enteric waterborne pathogenic bacteria and highly pathogenic protozoa such as *Cryptosporidium*, *Giardia*, and *N. fowleri.* However, less is known about the effectiveness of these disinfection processes against other FLA that are opportunistic pathogens or hosts for OPPPs (World Health Organization, 2017; Nisar et al., 2020a; National Health and Medical Research Council, 2022). Two studies from France examined the efficacy of chlorine, monochloramine, and chlorine dioxide against three environmental strains of *Acanthamoeba* spp., one *Naegleria* spp. isolate from potable water, and a reference collection strain of *Hartmannella* (Dupuy et al., 2011; Dupuy et al., 2014). Several studies have also investigated the effectiveness of different potable water disinfectants against *Acanthamoeba* spp. (Cursons et al., 1980; Loret et al., 2005; Ahmad et al., 2012; Cervero-Aragó et al., 2015; Menacho et al., 2024a; Menacho et al., 2024b). These recent studies have also considered the role of endosymbiotic bacteria in influencing disinfection outcomes. Notably, FLA are not inherently more resistant due to their isolation from a biofilm; rather, their resistance is enhanced when they are integrated into biofilms, where the biofilm matrix provides both physical and chemical protection (Nisar et al., 2022). However, only few studies have investigated the effectiveness of disinfection procedures against other environmental FLA associated with potable water or plumbing biofilms.

This study aimed to investigate and compare the effectiveness of different chemical disinfection processes and thermal disinfection (heat shock) against a range of FLA isolated from Australian domestic and hospital potable water and biofilm samples. The efficacy of thermal disinfection (70 °C), sodium hypochlorite, hydrogen peroxide, benzalkonium chloride, and 2-methyl-4-isothiazolin-3-one was tested against environmental isolates of *Acanthamoeba* DB1, *Allovahlkampfia* DS1, *Stenamoeba* DS1, and *V. vermiformis,* which have also been demonstrated to act as hosts for *Legionella.* The findings from this study will inform future disinfection protocols aimed at improving the management of FLA and OPPPs within engineered potable water systems.

## 2 Materials and methods

### 2.1 Amoeba strains and culturing conditions

This study used four different FLA strains previously isolated from engineered water systems (Nisar et al., 2022): *Acanthamoeba* DB1 (domestic tap faucet biofilm), *Allovahlkampfia* DS1 (domestic shower water), *Stenamoeba* DS1 (domestic shower water), and *Vermamoeba vermiformis* HB7 (hospital basin water), as well as *Acanthamoeba polyphaga* (ATCC^®^ 30461). Environmental isolates had their 18S rDNA region sequenced to confirm identification at the genus level (Nisar et al., 2022). Pure cultures of each FLA were maintained on 1.5% non-nutrient agar (Eco-NNA: CM0003, Oxoid Ltd.) plates supplemented with heat-inactivated (55 °C for 45 min) *Escherichia coli* (ATCC^®^ 700891). The plates were incubated under aerobic conditions at 25 °C for 10 days. Amoebic growth was monitored daily under an inverted light microscope (AMEFC4300, EVOS™ FL, Thermo Fisher Scientific, Waltham, Massachusetts, USA).

### 2.2 Trophozoite and cyst production

To assess trophozoites, FLA grown on Eco-NNA plates were harvested in 1X Page’s saline (120 mg NaCl, 4 g MgSO_4_.5H_2_O, 4 mg CaCl_2_.2H_2_O, 142 mg Na_2_HPO_4_, and 136 mg KH_2_PO_4_ per liter distilled water, pH 6.8 ± 0.2) and incubated under aerobic conditions at 25 °C for 3 h. For cyst assessment, amoeba encystment was induced by incubating plates for an additional 10 days at 30 °C to enhance encystment efficiency (Khan, 2006). The cysts were then harvested in 1X Page’s saline, washed twice, and incubated for 10 h in 3% HCl solution to kill any residual trophozoites and immature cysts (Critchley and Bentham, 2009). The trophozoites and cysts produced by the two methods described above were harvested in Tris-buffered saline (TBS) with a pH of 7.4 (Cat. No. T5030, Merck Pty. Ltd.) and were then enumerated using the trypan blue dye exclusion assay (Section 2.3).

### 2.3 Amoeba viability assays

The viability of amoeba trophozoites and cysts was determined using the trypan blue dye exclusion assay. Briefly, a 0.4% trypan blue solution (Cat. No. T8154, Merck Pty. Ltd.) and an aliquot of amoeba suspension were mixed in a 96-well plate at a 1:1 volume ratio (40 μL each) and incubated at room temperature for 5–10 min. This mixture was then visualized using an inverted light microscope (AMEFC4300, EVOS™ FL, Thermo Fisher Scientific). Trophozoites and cysts were enumerated using a Neubauer counting chamber (BLAUBRAND^®^), and both the stained (dead) and unstained (viable) cells were counted. The viability of the surviving amoeba cysts was also monitored by assessing their ability to transform into trophozoites (Strober, 2015). Briefly, the amoeba cysts suspended in 1X Page’s saline were inoculated into 96-well TC-treated microplates (Cat. No. CLS3595, Merck Pty. Ltd.) and incubated at 25 °C for 3–6 h. The growth of trophozoites was monitored under an inverted light microscope. For hemocytometer-based enumeration, the theoretical Limit of detection (LOD). was calculated from the minimum countable unit (one cell) over the counted chamber volume, adjusted for the 1:1 trypan blue dilution. Using a Neubauer chamber (depth 0.1 mm; 1 large square = 1 mm^2^ = 1 × 10^−4^ mL), four large squares per chamber on both sides were counted (10 squares total; 0.001 mL). Thus, an LOD was approximately 1 cell / 0.001 mL × 2 (dilution factor), which was equal to 2,000 cells/mL. The results below this threshold were treated as non-detects.

### 2.4 Physical and chemical disinfection procedures

The disinfection efficacy of seven different physical and chemical procedures was assessed based on the ASTM E645-18: standard test method for the evaluation of microbicides used in cooling towers (American Society for Testing and Materials International, 2018). Briefly, FLA cultures were exposed to disinfectants in bench-scale test systems consisting of sterile 96-well plates containing defined volumes of water and disinfectant under controlled laboratory conditions. All chemical disinfection assays were performed at 25 °C for a contact time of 1 h, unless otherwise stated. No additional ammonia or nitrogen source was present in the test systems; therefore, chloramine formation did not occur, and disinfectant activity reflected free chlorine. Temperature within each test system was monitored throughout the exposure period using a calibrated thermometer to ensure experimental consistency. Following exposure, viable FLA were quantified, and log reductions were calculated relative to untreated controls to determine disinfectant efficacy. All treatments were performed as technical replicates using the same prepared FLA suspension for each disinfectant condition.

### 2.5 Chemical disinfection procedures

The chemical disinfectants and concentrations tested in this study are representative of those typically used in the routine operation of engineered water systems and cooling towers, and are therefore contextually relevant to real-world applications. While sodium hypochlorite and chlorine dioxide are directly relevant to potable water treatment, benzalkonium chloride and 2-methyl-4-isothiazolin-3-one were included as comparative treatments due to their common application in cooling towers and industrial water circuits. Their inclusion provides useful context for evaluating amoeba disinfection across a broader range of engineered water systems. This included sodium hypochlorite (0–10 mg/L free chlorine, a range consistent with operational and regulatory limits for potable water systems; Cat. No. 425044, Merck Pty. Ltd.), chlorine dioxide (0–10 mg/L, CleanOxide^®^ tablets, Natural Water Solutions, Australia), benzalkonium chloride (0–50 mg/L, Cat. No. 12060, Merck Pty. Ltd.), hydrogen peroxide (0–5%, Cat. No. 216763, Merck Pty. Ltd.), and 2-methyl-4-isothiazolin-3-one (0–200 mg/L, Cat. No. 725765, Merck Pty. Ltd.). The concentrations of free chlorine in the chlorine-based disinfectants, i.e., sodium hypochlorite and chlorine dioxide, were confirmed using Mobile WaterLink^®^ Spin Touch^®^, SpinDisk™ Reagent Cartridge Single Use Treated Water Series DW13 (LaMotte Pacific Pty. Ltd.). Disinfection stock solutions of known concentrations were prepared according to the manufacturer’s specifications, and experimental concentrations were achieved by serial dilution in sterile water immediately prior to use. The efficacy of the disinfectants against each FLA was assessed by adding 10^6^ trophozoites or cysts/mL in 100 μL volumes into individual wells of a 96-well microtiter plate containing 100 μL of disinfectant at the desired test concentration. A relatively high starting concentration (10^6^ cells/mL) was chosen to enable quantification of multiple log reductions in viability. The plates were incubated at 25 °C for 1 h. Following exposure, 200 μL of Dey-Engley neutralizing broth (Cat. No. R453042, Remel Pty. Ltd.), equivalent to two times the combined volume of the sample and disinfectant, was added to each well to neutralize residual chemical disinfectant. Dey-Engley neutralizing broth was chosen for its ability to neutralize a broad spectrum of disinfectants, including quaternary ammonium compounds, phenolics, iodine, chlorine, aldehydes, and formaldehyde (Park and Chen, 2011). The trophozoites and cysts were washed with TBS, and cell suspensions were enumerated as described above using the trypan blue dye exclusion assay (Section 2.2).

### 2.6 Thermal disinfection procedure

The efficacy of thermal disinfection against FLA was assessed. Amoeba trophozoites and cysts (≈ 10^6^ cells/mL) in 1.5-mL Eppendorf tubes were placed in a calibrated water bath maintained at 70 ± 0.5 °C, with the temperature verified before and after each time point using a digital thermometer. The tubes were removed after 0, 1, 3, 5, 10, 20, 30, 45, and 60 min and immediately cooled on ice to halt further heat exposure. The viability of the thermally disinfected trophozoites and cysts was then enumerated as described above (Section 2.3).

### 2.7 Kinetic modeling of free-living amoeba disinfection

Non-linear regression modeling of disinfection kinetics was performed using the nls function in R (version 4.4.1). The viability data (log₁₀ reductions) of free-living amoebae exposed to different disinfectants over time or across concentration gradients were fitted to an exponential decay model to estimate initial viability (N₀) and decay rate constants (k). This approach enabled the quantitative comparison of disinfection efficacy among amoeba species and treatments.

## 3 Results

**Figure 1:**
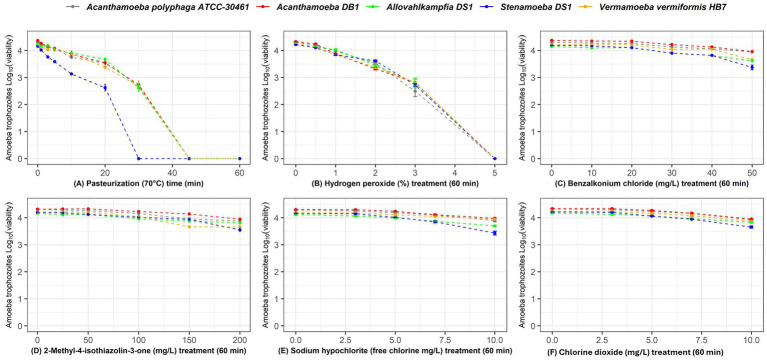
Survival of *Acanthamoeba polyphaga* ATCC^®^ 30461™ (gray), *Acanthamoeba* DB1 (red), *Allovahlkampfia* DS1 (green), *Stenamoeba* DS1 (blue), and *Vermamoeba vermiformis* HB7 (yellow) trophozoites when exposed to increasing concentrations/time for each disinfectant. **(A)** Thermal disinfection (70 °C) at 0, 1, 3, 5, 10, 20, 30, 45, and 60 min; **(B)** 0, 1, 2, 3, 4, and 5% hydrogen peroxide for 60 min; **(C)** 0 mg/L, 25 mg/L, 50 mg/L, 100 mg/L, 150 mg/L, and 200 mg/L 2-methyl-4-isothiazolin-3-one for 60 min; **(D)** 0 mg/L, 10 mg/L, 20 mg/L, 30 mg/L, 40 mg/L, and 50 mg/L of benzalkonium chloride for 60 min; **(E)** sodium hypochlorite at 0 mg/L, 3 mg/L, 5 mg/L, 7 mg/L, and 10 mg/L free chlorine for 60 min; and **(F)** chlorine dioxide at 0 mg/L, 3 mg/L, 5 mg/L, 7 mg/L, and 10 mg/L for 60 min. Data are presented as mean log_10_ viable cells/mL ± SD.

**Figure 2:**
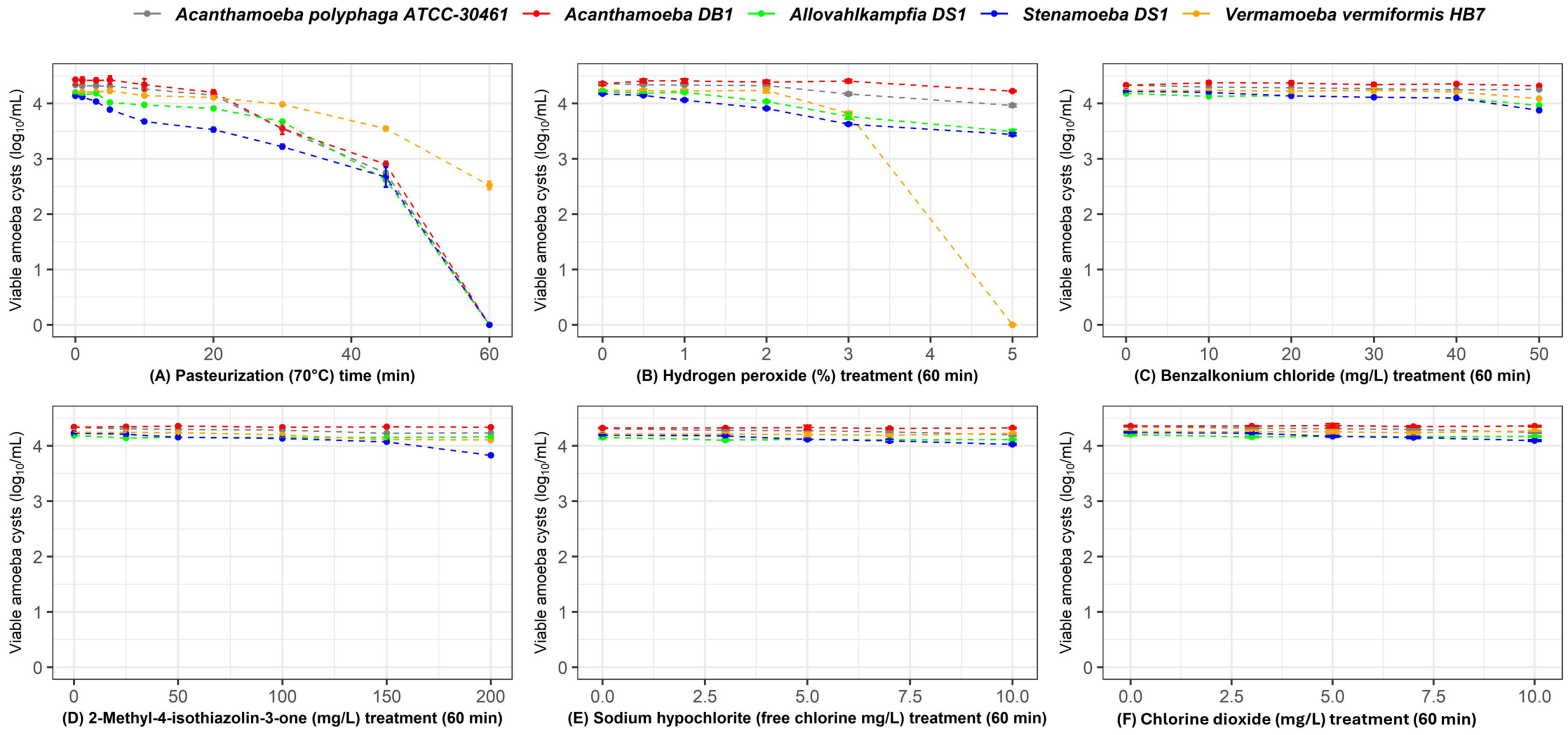
Survival of *Acanthamoeba polyphaga* ATCC^®^ 30461™ (gray), *Acanthamoeba* DB1 (red), *Allovahlkampfia* DS1 (green), *Stenamoeba* DS1 (blue), and *Vermamoeba vermiformis* HB7 (yellow) cysts when exposed to increasing concentrations/time for each disinfectant. **(A)** Thermal disinfection (70 °C) at 0, 1, 3, 5, 10, 20, 30, 45, and 60 min; **(B)** 0, 1, 2, 3, 4, and 5% hydrogen peroxide for 60 min; **(C)** 0 mg/L, 25 mg/L, 50 mg/L, 100 mg/L, 150 mg/L, and 200 mg/L 2-methyl-4-isothiazolin-3-one for 60 min; **(D)** 0 mg/L, 10 mg/L, 20 mg/L, 30 mg/L, 40 mg/L, and 50 mg/L of benzalkonium chloride for 60 min; **(E)** sodium hypochlorite at 0 mg/L, 3 mg/L, 5 mg/L, 7 mg/L, and 10 mg/L free chlorine for 60 min; and **(F)** chlorine dioxide at 0 mg/L, 3 mg/L, 5 mg/L, 7 mg/L, and 10 mg/L for 60 min. Data are presented as mean log_10_ viable cells/mL ± SD.

The efficacy of the different disinfectants against the trophozoites and cysts of *Acanthamoeba* DB1, *Allovahlkampfia* DS1, *Stenamoeba* DS1, and *V. vermiformis* HB7 isolated from Australian potable water and *A. polyphaga* ATCC^®^ 30461™ is shown in Figures 1, 2. The log_10_ reductions and percentage reductions for each disinfectant, time, concentration, and FLA are also presented in Supplementary Tables S1–S8 and Supplementary Figures S1, S2.

The first-order decay model revealed clear differences in disinfection efficacy across treatments, amoeba species, and life stages (Supplementary Tables S7, S8). Thermal disinfection at 70 °C was the most effective treatment overall, with consistent decay rates for trophozoites (k = 0.0289–0.0488) and cysts (k ≈ 0.017). A 45-min exposure was sufficient to eliminate all trophozoites, while a 60-min exposure effectively reduced cyst viability except for *V. vermiformis* HB7, which showed notable resistance. Hydrogen peroxide, tested up to 5% (a higher concentration than typically applied in operational settings) to enable comparison across disinfectants under the same 1-h contact time, was also highly effective against trophozoites (k = 0.255–0.272) but less so against cysts (k ≈ 0.03–0.06), except for *V. vermiformis* HB7, which displayed the greatest sensitivity among cysts. These findings highlight species-specific differences in susceptibility and support thermal disinfection and hydrogen peroxide as preferred methods for controlling free-living amoebae in water systems.

By contrast, benzalkonium chloride, 2-methyl-4-isothiazolin-3-one, sodium hypochlorite, and chlorine dioxide were largely ineffective, with none achieving >1 log₁₀ reduction against trophozoites or cysts at the maximum concentrations tested. Cysts were consistently more resistant than trophozoites, although *Stenamoeba* DS1 cysts were slightly more sensitive than those of other species. Overall, trophozoites were more sensitive than cysts across for all disinfectants and FLA tested (Figures 1, 2; Supplementary Tables S1–S6), as reflected in the kinetic decay rates, which were generally higher for trophozoites (k ≈ 0.009–0.014) than cysts (k ≈ 0.0001–0.0036) across treatments.

These results highlight pronounced species- and life-stage-specific differences in susceptibility and demonstrate that thermal disinfection and hydrogen peroxide are the only treatments tested with practical potential for controlling free-living amoebae in water systems.

## 4 Discussion

The presence of FLA in drinking water distribution systems and plumbing biofilms poses a public health risk due to their ability to act as opportunistic pathogens or as hosts for other OPPPs (Cateau et al., 2014; Balczun and Scheid, 2017; Fan et al., 2024). This study provides new evidence on the relative efficacy of disinfection strategies against environmental FLA isolated from Australian potable water and plumbing biofilms. Thermal disinfection and hydrogen peroxide were the most effective disinfection methods, with thermal disinfection showing the highest log reductions of both trophozoites and cysts. This supported previous findings from Cervero-Aragó et al. (2015) that found that thermal treatment (70 °C) was a more effective disinfectant compared with chlorine against an environmental strain and the reference strain *A. castellanii* CCAP 1534/2, indicating that these results may be broadly applicable across different species and strains of FLA. Thermal disinfection at 70 ^°^C for 45–60 min was necessary to achieve a 4 log reduction of trophozoites, with the shorter duration (10–30 min) resulting in only partial inactivation (Supplementary Table S1). Interestingly, despite trophozoites being the metabolically active form of FLA, they showed notable resistance to several disinfection methods. This may be due to physiological stress responses, such as encystment initiation or upregulation of protective enzymes, which can occur rapidly upon exposure to adverse conditions (Samba-Louaka et al., 2023). Such responses may reduce susceptibility to oxidative or chemical damage, potentially explaining the limited inactivation observed with certain treatments in this study. Heat shock or thermal disinfection is a method used routinely to disinfect building plumbing systems (Bédard et al., 2016; Ji et al., 2018). It involves raising the hot water temperature to 71–77 °C so that the temperature reaches at least 65 °C at the outlets, which are then flushed for 10–30 min (United States Environmental Protection Agency, 2016). This disinfection method is commonly used in hospitals and aged care facilities, as it does not require additional infrastructure. However, our findings indicate that the current implementation protocols may not achieve effective inactivation of environmental FLA, as 10–30 min at 70 °C resulted in only a 0.5–4 log reduction of trophozoites and <1 log reduction in cysts (Supplementary Table S1). This discrepancy between routine practice and disinfection efficacy demonstrates a need to re-evaluate standard thermal disinfection protocols, particularly in high-risk settings. Although thermal disinfection was the most effective method in the present study, it is generally unachievable to reach and maintain 70 °C with domestic water systems, and as such, this would only be an option for commercial and healthcare buildings (Carlson et al., 2020). Although the bench-scale experimental systems used do not fully replicate the complex hydrodynamic and biofilm conditions of drinking water distribution networks, they provide controlled conditions to assess the relative resistance of amoebae to thermal disinfection. This controlled approach allows for direct comparison across species and treatments, providing valuable foundational data. Future studies are needed to explore innovative building and plumbing designs that could facilitate delivery of this thermal disinfection process.

Hydrogen peroxide is an oxidizing agent approved for disinfection in many countries for a range of applications (Casini et al., 2017; Girolamini et al., 2019). A recent review identified it as one of the most promising, yet least studied, disinfectants for *Legionella* spp. control (Stavrou et al., 2020). In this study, hydrogen peroxide also showed strong efficacy against FLA. A previous study conducted by Hoogenkamp et al. (2023) demonstrated that low-dose hydrogen peroxide (0.02% for 5 h) did not kill the *V. vermiformis* but did detach amoeba from the biofilms, facilitating their removal from the system. The present study supports these findings and extends the research by quantifying the efficacy of hydrogen peroxide under controlled conditions against both trophozoites and cysts of multiple environmental FLA genera. Importantly, cyst viability was assessed post-treatment using trypan blue exclusion and excystation assays to confirm that no viable amoebae remained following exposure, ensuring that the observed reductions were not solely due to detachment or encystation. Despite its promise, hydrogen peroxide is not routinely used as a disinfectant for drinking water systems, and regulatory guidelines on safe residual levels are lacking (Silva and Sabogal-Paz, 2022). Its efficacy is influenced by pH, temperature, and organic content, which underscores the need for further field-based studies and standardized protocols to be developed before broader adoption (Girolamini et al., 2019).

Chlorine is the most common chemical disinfectant used to treat drinking water (Ngwenya et al., 2012). In this study, sodium hypochlorite, which provides free chlorine, was relatively ineffective against FLA. At 10 mg/L, trophozoites showed <0.5 log₁₀ reduction for the majority of strains except for *Stenamoeba* DS1 (0.73 log₁₀), whereas cysts were largely unaffected (<0.2 log₁₀ reduction; Supplementary Table S5, Figures 1, 2E). This limited efficacy is consistent with previous reports, which also found higher susceptibility of trophozoites compared with cysts, although overall log reductions remained low under comparable free chlorine exposures (Cervero-Aragó et al., 2015). However, simply increasing the chlorine concentration to improve the disinfection efficacy is not feasible, as disinfection by-products may exceed national drinking water quality guidelines (National Health and Medical Research Council, 2016). This research highlights the resilience of FLA cysts and trophozoites to conventional chlorine-based disinfection, as even high concentrations of chlorine or chlorine dioxide failed to achieve 1-log reductions. These findings indicate that reliance on chlorine alone is insufficient for effective FLA control, emphasizing the need for alternative or supplementary disinfection strategies.

The commonly used cooling tower biocides benzalkonium chloride and methylisothiazolinone showed no significant effects on the FLA studied, even at elevated concentrations (50 mg/L). Neither biocide showed appreciable reductions in concentrations of either trophozoites or cysts. These compounds were included because they are widely used for biofilm control in industrial water systems (e.g., cooling towers) and have reported antimicrobial activity against bacteria and fungi; however, their efficacy against free-living amoebae remains limited. By contrast, studies in the ophthalmic field have reported amoebicidal activity of benzalkonium chloride at very low concentrations against *Acanthamoeba* strains *in vitro*. For example, 0.003% benzalkonium chloride exhibited concentration- and time-dependent inhibitory effects on *Acanthamoeba* spp.(Tu et al., 2013). In our study, however, even much higher concentrations of benzalkonium chloride (50 mg/L) in suspension were ineffective against both trophozoites and cysts. This suggests that the efficacy of BAC is highly dependent on strain, physiological state, and environmental context, and that the results from clinical settings cannot be directly extrapolated to water systems. The use of these disinfectants in potable water is problematic, as they must be completely flushed from the system before it can be returned to normal use. Overall, the presented data indicate that these chemicals are not suitable candidates for amoeba control in potable water systems.

Within drinking water distribution systems, FLA will frequently contain intracellular bacteria, including human pathogens (Ashbolt, 2015). Previous research has shown that the presence of intracellular bacteria does not affect the efficacy of a disinfectant against amoeba (Dupuy et al., 2011; He et al., 2021). A previous study showed that the disinfection efficacy of chlorine and UV against *Dictyostelium discoideum* containing intracellular *Paraburkholderia agricolaris* was not affected by the intracellular presence of *P. agricolaris.* However, the disinfection methods were more effective against planktonic *P. agricolaris* compared with intracellular *P. agricolaris* (He et al., 2021). Similarly, a study demonstrated that monochloramine was equally effective against free and amoeba-associated *L. pneumophila*, whereas chlorine and chlorine dioxide were less effective against intracellular *L. pneumophila* compared with their efficacy against planktonic free-living cells (Dupuy et al., 2011). Although the present study did not quantify co-occurring pathogens such as *Legionella* spp., the data generated provide a novel and foundational understanding of the relative effectiveness of multiple disinfectants against environmental FLA isolated from both water and biofilms, including species not commonly studied such as *Allovahlkampfia* DS1 and *Stenamoeba* DS1. Importantly, biofilm-associated pathogens such as *L. pneumophila* are also more resistant to chlorine- and chloramine-based drinking water disinfectants than planktonic cells (Cooper and Hanlon, 2010; Buse Helen et al., 2019). In particular, the differences observed between amoebae of different genera in our study may be explained by variations in cyst wall composition, thickness, and permeability, as well as differences in trophozoite physiology, stress tolerance, and metabolic activity. For example, *Acanthamoeba* spp. are known to produce highly resistant cyst walls containing cellulose and other robust polymers, whereas *V. vermiformis* cysts are generally considered more permeable to disinfectants (Fouque et al., 2012). These structural and physiological distinctions may contribute to the differences in susceptibility observed across genera. Recent studies have highlighted the complex interactions between free-living amoebae, intracellular pathogens, and disinfectant efficacy, underscoring the importance of considering these relationships when evaluating water treatment strategies (Menacho et al., 2024b). Importantly, it is necessary to study a diverse range of amoebae species because even closely related amoebae within the same genus can exhibit markedly different disinfection susceptibilities and behaviors, which has significant implications for effective control strategies (Johnston et al., 2009). Our findings provide practical insights for water treatment professionals, informing optimized disinfection strategies tailored to target the most resistant FLA species and forms commonly found in potable water systems. While this study demonstrates that thermal disinfection and hydrogen peroxide can effectively reduce amoeba viability under controlled laboratory conditions, it is recognized that real-world applications often fall short of achieving these ideal disinfection levels due to operational limitations. Consequently, the persistence of FLA in water systems remains a concern. An additional limitation of this study is that disinfection treatments were assessed using technical replicates only, without independent biological replicates, which may affect reproducibility across separate amoeba preparations. Future studies can build on these findings and the methods established to explore the interactions between amoebae, disinfectants, and intracellular pathogens. This research will help clarify whether the disinfectant efficacy observed against FLA alone is predictive of broader control efficacy against amoeba-associated opportunistic pathogens.

## 5 Conclusion

This study showed that thermal disinfection and hydrogen peroxide were the most effective disinfectants against FLA, particularly trophozoites, isolated from drinking water distribution systems. These findings provide novel evidence for the relative efficacy of different disinfection strategies against environmental FLA and establish a foundation for further investigation. Future research is needed to explore the practical implementation of these disinfection strategies, as well as the development of alternative or complementary strategies. Such studies will support improved water quality management and contribute to public health protection by reducing risks directly associated with pathogenic FLA and limiting their role as hosts for intracellular opportunistic pathogens.

## Data Availability

The original contributions presented in the study are included in the article/Supplementary material, further inquiries can be directed to the corresponding author.
