# Supplementary material for: Efficacy of physical and chemical disinfection against clinically relevant free-living amoebae isolated from drinking water and plumbing biofilms
Source: Front Microbiol. 2025 Nov 28;16:1654984. doi: 10.3389/fmicb.2025.1654984 (PMC12698374; doi:10.3389/fmicb.2025.1654984)
Supplement: Supplementary file 1 [file Supplementary_file_1.docx]

Supplementary Material

Table S1: Disinfection efficacy (log_10_ reduction) of thermal disinfection (70^o^C thermal treatment) under varying exposure times (min) against *Acanthamoeba* DB1, *Allovahlkampfia* DS1, *Stenamoeba* DS1, and *Vermamoeba vermiformis* HB7 isolated from Australian potable water and *Acanthamoeba polyphaga* ATCC® 30461™ trophozoites and cysts.

| Amoebae | Exposure (min) | Thermal disinfection (70^o^C thermal treatment) disinfection efficacy | | | |
| --- | --- | --- | --- | --- | --- |
|  |  | Trophozoites | | Cysts | |
|  |  | Log10 reduction | ±SD | Log10 reduction | ±SD |
| *Acanthamoeba polyphaga* ATCC-30461 | 0 | 0.00 | 0.01 | 0.00 | 0.01 |
|  | 1 | 0.10 | 0.01 | 0.02 | 0.01 |
|  | 3 | 0.18 | 0.01 | 0.02 | 0.00 |
|  | 5 | 0.22 | 0.01 | 0.02 | 0.00 |
|  | 10 | 0.55 | 0.01 | 0.07 | 0.01 |
|  | 20 | 0.76 | 0.03 | 0.18 | 0.01 |
|  | 30 | 1.56 | 0.13 | 0.79 | 0.05 |
|  | 45 | 4.30 | 0.00 | 1.59 | 0.13 |
|  | 60 | 4.30 | 0.00 | 4.34 | 0.00 |
| *Acanthamoeba* DB1 | 0 | 0.00 | 0.04 | 0.00 | 0.09 |
|  | 1 | 0.10 | 0.01 | 0.01 | 0.06 |
|  | 3 | 0.22 | 0.02 | 0.01 | 0.04 |
|  | 5 | 0.29 | 0.01 | 0.01 | 0.07 |
|  | 10 | 0.50 | 0.10 | 0.09 | 0.11 |
|  | 20 | 0.82 | 0.10 | 0.23 | 0.04 |
|  | 30 | 1.65 | 0.12 | 0.88 | 0.10 |
|  | 45 | 4.37 | 0.00 | 1.53 | 0.05 |
|  | 60 | 4.37 | 0.00 | 4.43 | 0.00 |
| *Vermamoeba vermiformis* HB7 | 0 | 0.00 | 0.01 | 0.00 | 0.02 |
|  | 1 | 0.10 | 0.03 | 0.00 | 0.03 |
|  | 3 | 0.18 | 0.05 | 0.00 | 0.03 |
|  | 5 | 0.19 | 0.06 | 0.00 | 0.04 |
|  | 10 | 0.33 | 0.05 | 0.06 | 0.02 |
|  | 20 | 0.83 | 0.07 | 0.10 | 0.03 |
|  | 30 | 1.55 | 0.19 | 0.24 | 0.02 |
|  | 45 | 4.22 | 0.00 | 0.60 | 0.04 |
|  | 60 | 4.22 | 0.00 | 1.59 | 0.07 |
| *Allovahlkampfia* DS1 | 0 | 0.00 | 0.02 | 0.00 | 0.02 |
|  | 1 | 0.03 | 0.01 | 0.03 | 0.01 |
|  | 3 | 0.01 | 0.02 | 0.01 | 0.02 |
|  | 5 | 0.17 | 0.02 | 0.17 | 0.02 |
|  | 10 | 0.28 | 0.01 | 0.22 | 0.01 |
|  | 20 | 0.52 | 0.03 | 0.28 | 0.01 |
|  | 30 | 1.57 | 0.13 | 0.52 | 0.03 |
|  | 45 | 4.19 | 0.00 | 1.57 | 0.13 |
|  | 60 | 4.19 | 0.00 | 4.19 | 0.00 |
| *Stenamoeba* DS1 | 0 | 0.00 | 0.01 | 0.00 | 0.01 |
|  | 1 | 0.14 | 0.01 | 0.02 | 0.01 |
|  | 3 | 0.40 | 0.03 | 0.10 | 0.02 |
|  | 5 | 0.57 | 0.01 | 0.25 | 0.02 |
|  | 10 | 1.02 | 0.04 | 0.46 | 0.02 |
|  | 20 | 1.53 | 0.13 | 0.61 | 0.04 |
|  | 30 | 4.16 | 0.00 | 0.92 | 0.04 |
|  | 45 | 4.16 | 0.00 | 1.46 | 0.19 |
|  | 60 | 4.16 | 0.00 | 4.14 | 0.00 |

Table S2: Disinfection efficacy (log_10_ reduction) of varying concentrations (%) of Hydrogen peroxide (60 min exposure time) against *Acanthamoeba* DB1, *Allovahlkampfia* DS1, *Stenamoeba* DS1, and *Vermamoeba vermiformis* HB7 isolated from Australian potable water and *Acanthamoeba polyphaga* ATCC® 30461™ trophozoites and cysts.

| **Amoebae** | **Concentration (%)** | **Disinfection efficacy of Hydrogen peroxide (60 min exposure time)** | | | |
| --- | --- | --- | --- | --- | --- |
|  |  | **Trophozoites** | | **Cysts** | |
|  |  | **Log10 reduction** | **±SD** | **Log10 reduction** | **±SD** |
| *Acanthamoeba polyphaga* ATCC-30461 | 0 | 0.00 | 0.01 | 0.00 | 0.00 |
|  | 0.5 | 0.12 | 0.01 | 0.02 | 0.01 |
|  | 1 | 0.33 | 0.01 | 0.03 | 0.00 |
|  | 2 | 0.78 | 0.05 | 0.04 | 0.01 |
|  | 3 | 1.82 | 0.20 | 0.19 | 0.02 |
|  | 5 | 4.31 | 0.00 | 0.40 | 0.02 |
| *Acanthamoeba* DB1 | 0 | 0.00 | 0.01 | 0.00 | 0.03 |
|  | 0.5 | 0.09 | 0.01 | -0.05 | 0.03 |
|  | 1 | 0.45 | 0.00 | -0.05 | 0.04 |
|  | 2 | 1.01 | 0.04 | -0.03 | 0.03 |
|  | 3 | 1.51 | 0.11 | -0.05 | 0.02 |
|  | 5 | 4.33 | 0.00 | 0.13 | 0.01 |
| *Vermamoeba vermiformis* HB7 | 0 | 0.00 | 0.01 | 0.00 | 0.01 |
|  | 0.5 | 0.13 | 0.01 | 0.01 | 0.03 |
|  | 1 | 0.42 | 0.04 | 0.01 | 0.03 |
|  | 2 | 0.87 | 0.05 | 0.00 | 0.04 |
|  | 3 | 1.45 | 0.11 | 0.42 | 0.03 |
|  | 5 | 4.26 | 0.00 | 4.24 | 0.00 |
| *Allovahlkampfia* DS1 | 0 | 0.00 | 0.02 | 0.00 | 0.02 |
|  | 0.5 | 0.13 | 0.01 | 0.04 | 0.01 |
|  | 1 | 0.24 | 0.02 | 0.02 | 0.01 |
|  | 2 | 0.81 | 0.06 | 0.19 | 0.01 |
|  | 3 | 1.46 | 0.18 | 0.46 | 0.06 |
|  | 5 | 4.26 | 0.00 | 0.73 | 0.03 |
| *Stenamoeba* DS1 | 0 | 0.00 | 0.00 | 0.00 | 0.01 |
|  | 0.5 | 0.12 | 0.01 | 0.03 | 0.01 |
|  | 1 | 0.37 | 0.03 | 0.11 | 0.01 |
|  | 2 | 0.60 | 0.03 | 0.26 | 0.01 |
|  | 3 | 1.49 | 0.05 | 0.55 | 0.02 |
|  | 5 | 4.22 | 0.00 | 0.73 | 0.02 |

Table S3: Disinfection efficacy (log_10_ reduction) of varying concentrations (mg/L) of Benzalkonium chloride treatment (60 min exposure time) against *Acanthamoeba* DB1, *Allovahlkampfia* DS1, *Stenamoeba* DS1, and *Vermamoeba vermiformis* HB7 isolated from Australian potable water and *Acanthamoeba polyphaga* ATCC® 30461™ trophozoites and cysts.

| **Amoebae** | **Concentration (mg/L)** | **Disinfection efficacy of Benzalkonium chloride (60 mins exposure)** | | | |
| --- | --- | --- | --- | --- | --- |
|  |  | **Trophozoites** | | **Cysts** | |
|  |  | **Log10 reduction** | **±SD** | **Log10 reduction** | **±SD** |
| *Acanthamoeba polyphaga* ATCC-30461 | 0 | 0.00 | 0.00 | 0.00 | 0.00 |
|  | 10 | 0.03 | 0.01 | 0.03 | 0.01 |
|  | 20 | 0.05 | 0.00 | 0.04 | 0.00 |
|  | 30 | 0.17 | 0.01 | 0.06 | 0.01 |
|  | 40 | 0.24 | 0.02 | 0.08 | 0.02 |
|  | 50 | 0.36 | 0.04 | 0.07 | 0.01 |
| *Acanthamoeba* DB1 | 0 | 0.00 | 0.02 | 0.00 | 0.02 |
|  | 10 | 0.03 | 0.03 | -0.04 | 0.03 |
|  | 20 | 0.03 | 0.04 | -0.04 | 0.04 |
|  | 30 | 0.16 | 0.01 | -0.01 | 0.04 |
|  | 40 | 0.25 | 0.01 | -0.02 | 0.03 |
|  | 50 | 0.41 | 0.03 | 0.01 | 0.02 |
| *Vermamoeba vermiformis* HB7 | 0 | 0.00 | 0.01 | 0.00 | 0.01 |
|  | 10 | -0.02 | 0.02 | 0.01 | 0.03 |
|  | 20 | 0.00 | 0.02 | 0.01 | 0.03 |
|  | 30 | 0.17 | 0.02 | 0.00 | 0.04 |
|  | 40 | 0.16 | 0.02 | 0.02 | 0.01 |
|  | 50 | 0.56 | 0.04 | 0.15 | 0.01 |
| *Allovahlkampfia* DS1 | 0 | 0.00 | 0.03 | 0.00 | 0.02 |
|  | 10 | 0.06 | 0.01 | 0.05 | 0.01 |
|  | 20 | 0.05 | 0.01 | 0.04 | 0.01 |
|  | 30 | 0.25 | 0.03 | 0.07 | 0.02 |
|  | 40 | 0.33 | 0.02 | 0.08 | 0.01 |
|  | 50 | 0.54 | 0.02 | 0.22 | 0.02 |
| *Stenamoeba* DS1 | 0 | 0.00 | 0.01 | 0.00 | 0.01 |
|  | 10 | 0.02 | 0.01 | 0.02 | 0.01 |
|  | 20 | 0.09 | 0.01 | 0.08 | 0.01 |
|  | 30 | 0.29 | 0.03 | 0.11 | 0.02 |
|  | 40 | 0.37 | 0.02 | 0.12 | 0.01 |
|  | 50 | 0.82 | 0.09 | 0.34 | 0.02 |

Table S4: Disinfection efficacy (log_10_ reduction) of varying concentrations (mg/L) of 2-Methyl-4-isothiazolin-3-one treatment (60 min) against *Acanthamoeba* DB1, *Allovahlkampfia* DS1, *Stenamoeba* DS1, and *Vermamoeba vermiformis* HB7 isolated from Australian potable water and *Acanthamoeba polyphaga* ATCC® 30461™ trophozoites and cysts.

| **Amoebae** | **Concentration (mg/L)** | **Disinfection efficacy of 2-Methyl-4-isothiazolin-3-one (mg/L) treatment (60 min)** | | | |
| --- | --- | --- | --- | --- | --- |
|  |  | **Trophozoites** | | **Cysts** | |
|  |  | **Log10 reduction** | **±SD** | **Log10 reduction** | **±SD** |
| Acanthamoeba polyphaga ATCC-30461 | 0 | 0.00 | 0.00 | 0.00 | 0.00 |
|  | 25 | 0.03 | 0.01 | 0.03 | 0.01 |
|  | 50 | 0.05 | 0.01 | 0.04 | 0.00 |
|  | 100 | 0.16 | 0.01 | 0.05 | 0.01 |
|  | 150 | 0.35 | 0.01 | 0.11 | 0.02 |
|  | 200 | 0.43 | 0.01 | 0.10 | 0.02 |
| Acanthamoeba DB1 | 0 | 0.00 | 0.02 | 0.00 | 0.02 |
|  | 25 | -0.01* | 0.00 | -0.01 | 0.00 |
|  | 50 | -0.01* | 0.02 | -0.02 | 0.03 |
|  | 100 | 0.09 | 0.00 | -0.10 | 0.01 |
|  | 150 | 0.18 | 0.01 | -0.01 | 0.01 |
|  | 200 | 0.37 | 0.04 | 0.00 | 0.02 |
| Vermamoeba vermiformis HB7 | 0 | 0.00 | 0.01 | 0.00 | 0.01 |
|  | 25 | 0.00 | 0.03 | 0.00 | 0.03 |
|  | 50 | 0.01 | 0.01 | 0.01 | 0.03 |
|  | 100 | 0.17 | 0.03 | 0.04 | 0.02 |
|  | 150 | 0.55 | 0.03 | 0.13 | 0.00 |
|  | 200 | 0.56 | 0.08 | 0.13 | 0.02 |
| Allovahlkampfia DS1 | 0 | 0.00 | 0.02 | 0.00 | 0.02 |
|  | 25 | 0.06 | 0.01 | 0.05 | 0.01 |
|  | 50 | 0.04 | 0.01 | 0.04 | 0.01 |
|  | 100 | 0.20 | 0.03 | 0.04 | 0.01 |
|  | 150 | 0.29 | 0.02 | 0.04 | 0.01 |
|  | 200 | 0.35 | 0.04 | 0.03 | 0.02 |
| Stenamoeba DS1 | 0 | 0.00 | 0.01 | 0.00 | 0.01 |
|  | 25 | 0.01 | 0.01 | 0.01 | 0.01 |
|  | 50 | 0.08 | 0.01 | 0.07 | 0.01 |
|  | 100 | 0.18 | 0.01 | 0.09 | 0.02 |
|  | 150 | 0.26 | 0.02 | 0.15 | 0.01 |
|  | 200 | 0.65 | 0.07 | 0.40 | 0.02 |

Table S5: Disinfection efficacy (log_10_ reduction) of varying concentrations (free chlorine mg/L) of sodium hypochlorite (60 min exposure) against *Acanthamoeba* DB1, *Allovahlkampfia* DS1, *Stenamoeba* DS1, and *Vermamoeba vermiformis* HB7 isolated from Australian potable water and *Acanthamoeba polyphaga* ATCC® 30461™ trophozoites and cysts.

| **Amoebae** | **Concentration of free chlorine (mg/L)** | **Disinfection efficacy of Sodium hypochlorite (free chlorine mg/L) treatment (60 min)** | | | |
| --- | --- | --- | --- | --- | --- |
|  |  | **Trophozoites** | | **Cysts** | |
|  |  | **Log10 reduction** | **±SD** | **Log10 reduction** | **±SD** |
| Acanthamoeba polyphaga ATCC-30461 | 0 | 0.00 | 0.00 | 0.00 | 0.00 |
|  | 3 | 0.03 | 0.02 | 0.03 | 0.01 |
|  | 5 | 0.10 | 0.01 | 0.04 | 0.00 |
|  | 7 | 0.18 | 0.01 | 0.06 | 0.01 |
|  | 10 | 0.39 | 0.05 | 0.11 | 0.02 |
| Acanthamoeba DB1 | 0 | 0.00 | 0.01 | 0.00 | 0.01 |
|  | 3 | 0.00 | 0.01 | 0.00 | 0.00 |
|  | 5 | 0.06 | 0.01 | -0.01 | 0.04 |
|  | 7 | 0.19 | 0.01 | 0.01 | 0.01 |
|  | 10 | 0.32 | 0.01 | 0.00 | 0.01 |
| Vermamoeba vermiformis HB7 | 0 | 0.00 | 0.01 | 0.00 | 0.01 |
|  | 3 | 0.00 | 0.04 | 0.00 | 0.03 |
|  | 5 | 0.08 | 0.04 | 0.01 | 0.03 |
|  | 7 | 0.13 | 0.01 | 0.23 | 0.02 |
|  | 10 | 0.23 | 0.02 | -0.01 | 0.01 |
| Allovahlkampfia DS1 | 0 | 0.00 | 0.03 | 0.00 | 0.03 |
|  | 3 | 0.06 | 0.01 | 0.05 | 0.01 |
|  | 5 | 0.13 | 0.02 | 0.04 | 0.01 |
|  | 7 | 0.25 | 0.03 | 0.05 | 0.01 |
|  | 10 | 0.43 | 0.02 | 0.05 | 0.01 |
| Stenamoeba DS1 | 0 | 0.00 | 0.00 | 0.00 | 0.01 |
|  | 3 | 0.02 | 0.01 | 0.02 | 0.01 |
|  | 5 | 0.15 | 0.01 | 0.08 | 0.01 |
|  | 7 | 0.32 | 0.03 | 0.10 | 0.02 |
|  | 10 | 0.73 | 0.07 | 0.17 | 0.01 |

Table S6: Disinfection efficacy (log_10_ reduction) of varying concentrations (mg/L) of chlorine dioxide (60 min exposure) against *Acanthamoeba* DB1, *Allovahlkampfia* DS1, *Stenamoeba* DS1, and *Vermamoeba vermiformis* HB7 isolated from Australian potable water and *Acanthamoeba polyphaga* ATCC® 30461™ trophozoites and cysts.

| **Amoebae** | **Concentration of free chlorine (mg/L)** | **Disinfection efficacy of Chlorine dioxide (mg/L) treatment (60 min)** | | | |
| --- | --- | --- | --- | --- | --- |
|  |  | **Trophozoites** | | **Cysts** | |
|  |  | **Log10 reduction** | **±SD** | **Log10 reduction** | **±SD** |
| *Acanthamoeba polyphaga* ATCC-30461 | 0 | 0.00 | 0.00 | 0.00 | 0.00 |
|  | 3 | 0.03 | 0.01 | 0.03 | 0.01 |
|  | 5 | 0.09 | 0.01 | 0.04 | 0.00 |
|  | 7 | 0.17 | 0.01 | 0.05 | 0.01 |
|  | 10 | 0.40 | 0.03 | 0.10 | 0.02 |
| *Acanthamoeba* DB1 | 0 | 0.00 | 0.01 | 0.00 | 0.01 |
|  | 3 | 0.00 | 0.00 | 0.00 | 0.00 |
|  | 5 | 0.07 | 0.02 | -0.01 | 0.03 |
|  | 7 | 0.17 | 0.01 | 0.01 | 0.01 |
|  | 10 | 0.39 | 0.01 | 0.00 | 0.01 |
| *Vermamoeba vermiformis* HB7 | 0 | 0.00 | 0.01 | 0.00 | 0.01 |
|  | 3 | 0.00 | 0.03 | 0.00 | 0.03 |
|  | 5 | 0.00 | 0.04 | 0.01 | 0.03 |
|  | 7 | 0.15 | 0.03 | 0.02 | 0.02 |
|  | 10 | 0.36 | 0.01 | -0.01 | 0.00 |
| *Allovahlkampfia* DS1 | 0 | 0.00 | 0.03 | 0.00 | 0.02 |
|  | 3 | 0.05 | 0.01 | 0.05 | 0.01 |
|  | 5 | 0.11 | 0.02 | 0.03 | 0.01 |
|  | 7 | 0.21 | 0.02 | 0.04 | 0.01 |
|  | 10 | 0.35 | 0.01 | 0.04 | 0.01 |
| *Stenamoeba* DS1 | 0 | 0.00 | 0.01 | 0.00 | 0.01 |
|  | 3 | 0.01 | 0.01 | 0.01 | 0.01 |
|  | 5 | 0.15 | 0.02 | 0.07 | 0.01 |
|  | 7 | 0.27 | 0.02 | 0.09 | 0.02 |
|  | 10 | 0.56 | 0.04 | 0.15 | 0.01 |

Table S7.

Estimated kinetic parameters for each amoeba species (cysts) under different disinfection treatments. Values represent the decay rate constant (k, min⁻¹ or per concentration unit as applicable) and initial viability (N₀), obtained by fitting the disinfection data to an exponential decay model in R (version 4.4.1).

| Amoebae | Treatment | N0 | k |
| --- | --- | --- | --- |
| *Acanthamoeba polyphaga* ATCC-30461 | Pasteurization (70°C) time (min) | 4.716895 | 1.70E-02 |
| *Acanthamoeba* DB1 | Pasteurization (70°C) time (min) | 4.815275 | 1.69E-02 |
| *Vermamoeba vermiformis* HB7 | Pasteurization (70°C) time (min) | 4.364305 | 6.11E-03 |
| *Allovahlkampfia*DS1 | Pasteurization (70°C) time (min) | 4.504102 | 1.65E-02 |
| Stenamoeba DS1 | Pasteurization (70°C) time (min) | 4.345207 | 1.75E-02 |
| *Acanthamoeba polyphaga* ATCC-30461 | Hydrogen peroxide (%) treatment (60 min) | 4.40306 | 1.88E-02 |
| *Acanthamoeba* DB1 | Hydrogen peroxide (%) treatment (60 min) | 4.4167 | 6.18E-03 |
| *Vermamoeba vermiformis* HB7 | Hydrogen peroxide (%) treatment (60 min) | 4.880486 | 2.00E-01 |
| *Allovahlkampfia*DS1 | Hydrogen peroxide (%) treatment (60 min) | 4.293856 | 3.99E-02 |
| Stenamoeba DS1 | Hydrogen peroxide (%) treatment (60 min) | 4.210418 | 4.19E-02 |
| *Acanthamoeba polyphaga* ATCC-30461 | Benzalkonium chloride (mg/L) treatment (60 min) | 4.320392 | 3.64E-04 |
| *Acanthamoeba* DB1 | Benzalkonium chloride (mg/L) treatment (60 min) | 4.359021 | 9.81E-05 |
| *Vermamoeba vermiformis* HB7 | Benzalkonium chloride (mg/L) treatment (60 min) | 4.262561 | 5.42E-04 |
| *Allovahlkampfia*DS1 | Benzalkonium chloride (mg/L) treatment (60 min) | 4.191604 | 8.31E-04 |
| Stenamoeba DS1 | Benzalkonium chloride (mg/L) treatment (60 min) | 4.255483 | 1.42E-03 |
| *Acanthamoeba polyphaga* ATCC-30461 | Sodium hypochlorite (free chlorine mg/L) treatment (60 min) | 4.316526 | 2.49E-03 |
| *Acanthamoeba* DB1 | Sodium hypochlorite (free chlorine mg/L) treatment (60 min) | 4.325759 | 1.35E-04 |
| *Vermamoeba vermiformis* HB7 | Sodium hypochlorite (free chlorine mg/L) treatment (60 min) | 4.207756 | -1.17E-04 |
| *Allovahlkampfia*DS1 | Sodium hypochlorite (free chlorine mg/L) treatment (60 min) | 4.138436 | 9.08E-04 |
| Stenamoeba DS1 | Sodium hypochlorite (free chlorine mg/L) treatment (60 min) | 4.210314 | 4.21E-03 |
| *Acanthamoeba polyphaga* ATCC-30461 | Chlorine dioxide (free chlorine mg/L) treatment (60 min) | 4.352477 | 2.24E-03 |
| *Acanthamoeba* DB1 | Chlorine dioxide (free chlorine mg/L) treatment (60 min) | 4.361225 | 1.23E-04 |
| *Vermamoeba vermiformis* HB7 | Chlorine dioxide (free chlorine mg/L) treatment (60 min) | 4.253759 | -1.04E-04 |
| *Allovahlkampfia*DS1 | Chlorine dioxide (free chlorine mg/L) treatment (60 min) | 4.192007 | 7.94E-04 |
| Stenamoeba DS1 | Chlorine dioxide (free chlorine mg/L) treatment (60 min) | 4.255346 | 3.65E-03 |

Table S8.

Estimated kinetic parameters for each amoeba species (cysts) under different disinfection treatments. Values represent the decay rate constant (k, min⁻¹ or per concentration unit as applicable) and initial viability (N₀), obtained by fitting the disinfection data to an exponential decay model in R (version 4.4.1).

| Amoebae | Treatment | N0 | k |
| --- | --- | --- | --- |
| *Acanthamoeba polyphaga* ATCC-30461 | Pasteurization (70°C) time (min) | 4.623476 | 0.0293444 |
| *Acanthamoeba* DB1 | Pasteurization (70°C) time (min) | 4.683509 | 0.0296154 |
| *Vermamoeba vermiformis* HB7 | Pasteurization (70°C) time (min) | 4.570497 | 0.0294551 |
| *Allovahlkampfia*DS1 | Pasteurization (70°C) time (min) | 4.614113 | 0.0289368 |
| Stenamoeba DS1 | Pasteurization (70°C) time (min) | 4.40021 | 0.0488392 |
| *Acanthamoeba polyphaga* ATCC-30461 | Hydrogen peroxide (%) treatment (60 min) | 4.814715 | 0.2724745 |
| *Acanthamoeba* DB1 | Hydrogen peroxide (%) treatment (60 min) | 4.790771 | 0.2663853 |
| *Vermamoeba vermiformis* HB7 | Hydrogen peroxide (%) treatment (60 min) | 4.715287 | 0.2598921 |
| *Allovahlkampfia*DS1 | Hydrogen peroxide (%) treatment (60 min) | 4.762902 | 0.2578375 |
| Stenamoeba DS1 | Hydrogen peroxide (%) treatment (60 min) | 4.701519 | 0.2547616 |
| *Acanthamoeba polyphaga* ATCC-30461 | Benzalkonium chloride (mg/L) treatment (60 min) | 4.350428 | 0.0017574 |
| *Acanthamoeba* DB1 | Benzalkonium chloride (mg/L) treatment (60 min) | 4.433376 | 0.0018985 |
| *Vermamoeba vermiformis* HB7 | Benzalkonium chloride (mg/L) treatment (60 min) | 4.314721 | 0.0023959 |
| *Allovahlkampfia*DS1 | Benzalkonium chloride (mg/L) treatment (60 min) | 4.216149 | 0.0026612 |
| Stenamoeba DS1 | Benzalkonium chloride (mg/L) treatment (60 min) | 4.311676 | 0.0038122 |
| *Acanthamoeba polyphaga* ATCC-30461 | Sodium hypochlorite (free chlorine mg/L) treatment (60 min) | 4.341651 | 0.0093874 |
| *Acanthamoeba* DB1 | Sodium hypochlorite (free chlorine mg/L) treatment (60 min) | 4.357179 | 0.0080836 |
| *Vermamoeba vermiformis* HB7 | Sodium hypochlorite (free chlorine mg/L) treatment (60 min) | 4.217915 | 0.0060079 |
| *Allovahlkampfia*DS1 | Sodium hypochlorite (free chlorine mg/L) treatment (60 min) | 4.167396 | 0.0108625 |
| Stenamoeba DS1 | Sodium hypochlorite (free chlorine mg/L) treatment (60 min) | 4.288488 | 0.01825 |
| *Acanthamoeba polyphaga* ATCC-30461 | Chlorine dioxide (free chlorine mg/L) treatment (60 min) | 4.384727 | 0.0092557 |
| *Acanthamoeba* DB1 | Chlorine dioxide (free chlorine mg/L) treatment (60 min) | 4.408749 | 0.0092107 |
| *Vermamoeba vermiformis* HB7 | Chlorine dioxide (free chlorine mg/L) treatment (60 min) | 4.296612 | 0.0087076 |
| *Allovahlkampfia*DS1 | Chlorine dioxide (free chlorine mg/L) treatment (60 min) | 4.211652 | 0.0087258 |
| Stenamoeba DS1 | Chlorine dioxide (free chlorine mg/L) treatment (60 min) | 4.301713 | 0.0139586 |


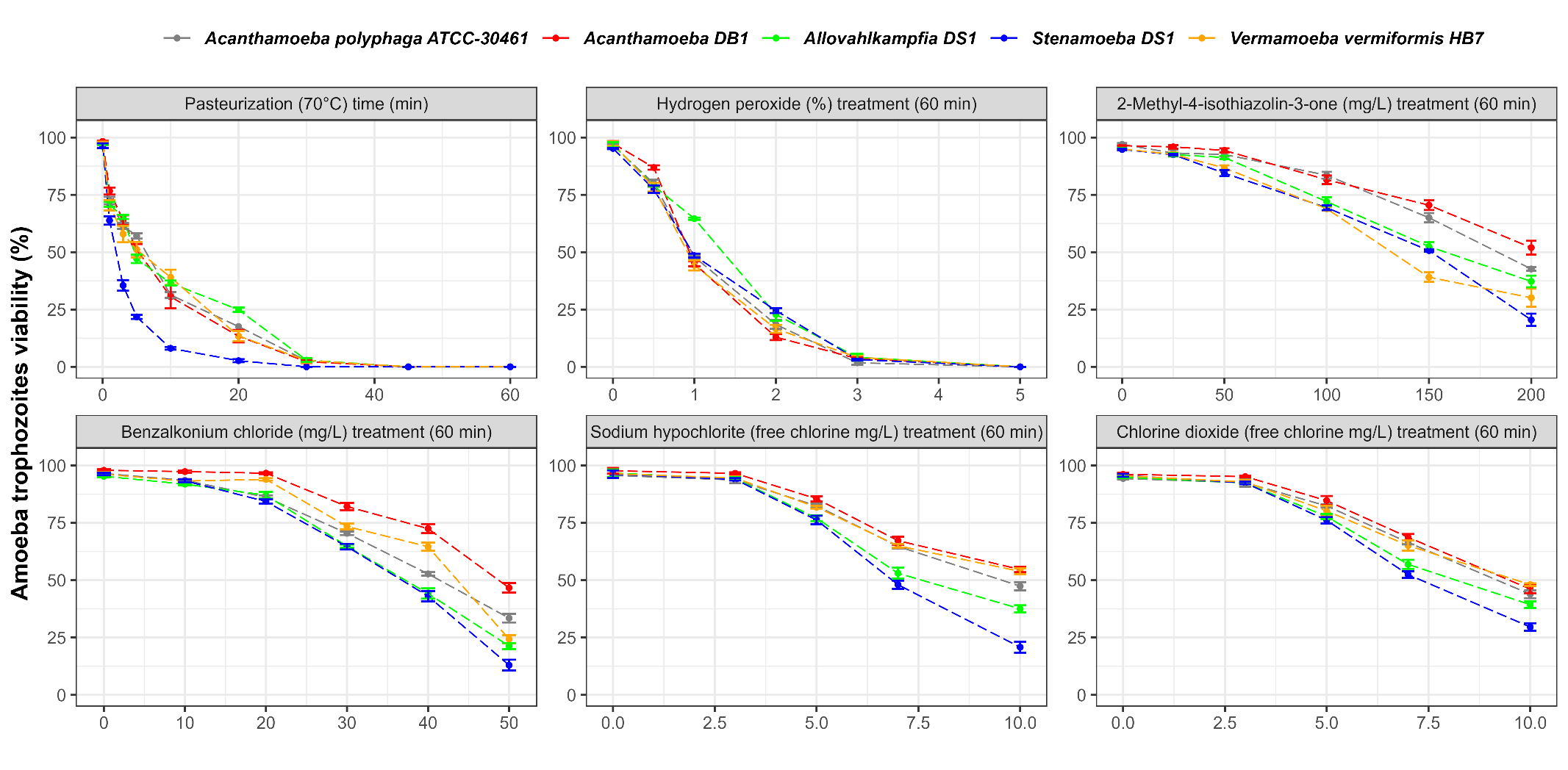


A)

B)

C)

D)

E)

F)

Chlorine dioxide (mg/L) treatment (60 min)

Figure S1: Percentage survival of *Acanthamoeba polyphaga* ATCC® 30461™ (grey) *Acanthamoeba* DB1 (red), *Allovahlkampfia* DS1 (green), *Stenamoeba* DS1 (blue), and *Vermamoeba vermiformis* HB7 (yellow) trophozoites when exposed to increasing concentrations/time for each disinfectant. A) thermal disinfection (70^o^C) at 0, 1, 3, 5, 10, 20, 30, 45, 60 min; B) 0, 1%, 2%, 3%, 4%, and 5% hydrogen peroxide for 60 mins; C) 0, 25 mg/L, 50 mg/L, 100 mg/L, 150 mg/L and 200 mg/L 2-Methyl-4-isothiazolin-3-one for 60 mins; D) 0, 10 mg/L, 20 mg/L, 30 mg/L, 40 mg/L and 50 mg/L of Benzalkonium chloride for 60 mins; E) sodium hypochlorite at 0, 3 mg/L, 5 mg/L, 7 mg/L and 10 mg/L free chlorine for 60 mins; F) chlorine dioxide at 0, 3 mg/L, 5 mg/L, 7 mg/L and 10 mg/L for 60 mins.


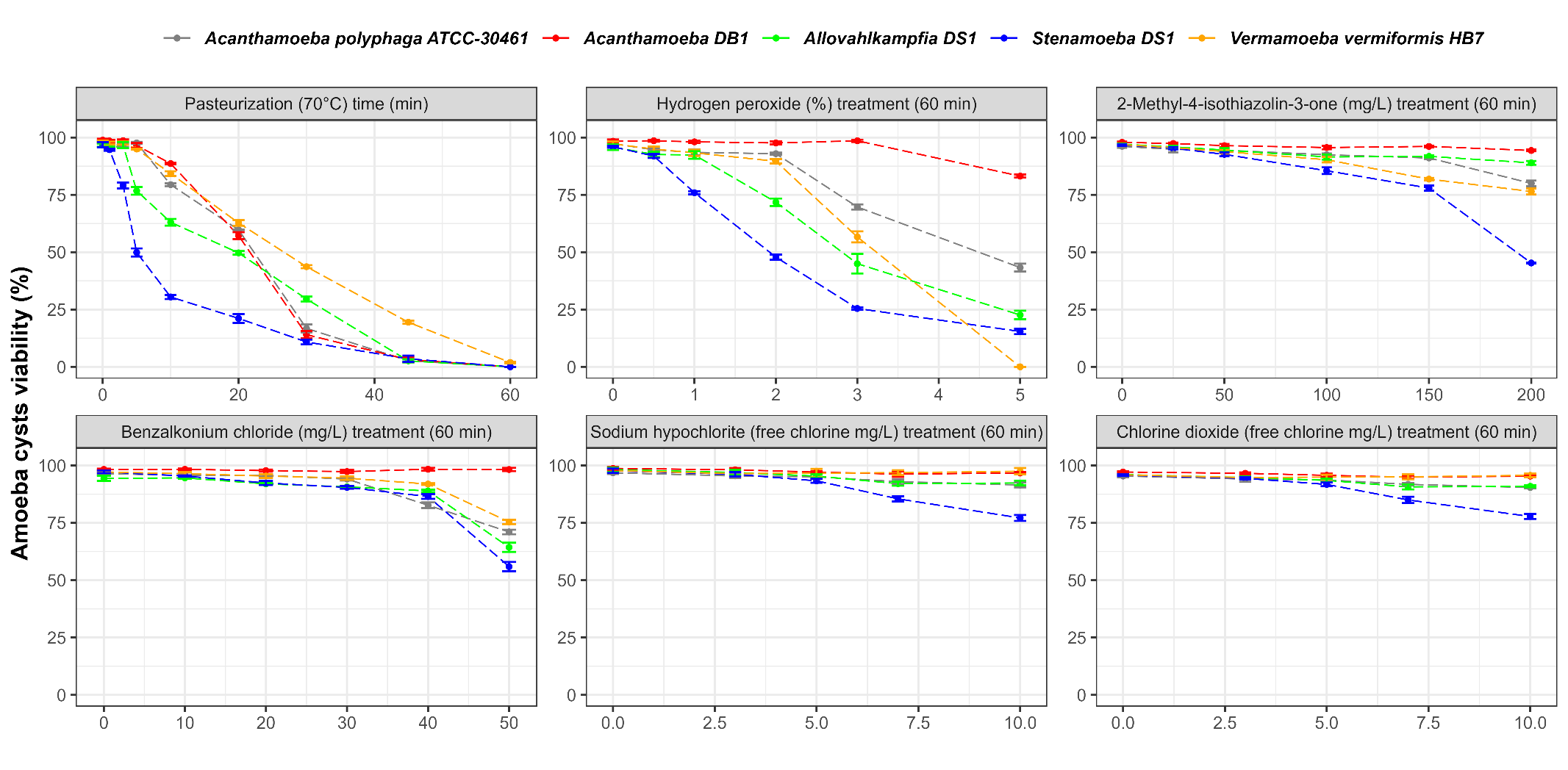


A)

B)

C)

D)

E)

F)

Chlorine dioxide (mg/L) treatment (60 min)

Figure S2: Percentage survival of *Acanthamoeba polyphaga* ATCC® 30461™ (grey) *Acanthamoeba* DB1 (red), *Allovahlkampfia* DS1 (green), *Stenamoeba* DS1 (blue), and *Vermamoeba vermiformis* HB7 (yellow) cysts when exposed to increasing concentrations/time for each disinfectant. A) thermal disinfection (70^o^C) at 0, 1, 3, 5, 10, 20, 30, 45, 60 min; B) 0, 1%, 2%, 3%, 4%, and 5% hydrogen peroxide for 60 mins; C) 0, 25 mg/L, 50 mg/L, 100 mg/L, 150 mg/L and 200 mg/L 2-Methyl-4-isothiazolin-3-one for 60 mins; D) 0, 10 mg/L, 20 mg/L, 30 mg/L, 40 mg/L and 50 mg/L of Benzalkonium chloride for 60 mins; E) sodium hypochlorite at 0, 3 mg/L, 5 mg/L, 7 mg/L and 10 mg/L free chlorine for 60 mins; F) chlorine dioxide at 0, 3 mg/L, 5 mg/L, 7 mg/L and 10 mg/L for 60 mins.
